# Circulating adenosine increases during human experimental endotoxemia but blockade of its receptor does not influence the immune response and subsequent organ injury

**DOI:** 10.1186/cc9400

**Published:** 2011-01-06

**Authors:** Bart P Ramakers, Niels P Riksen, Petra van den Broek, Barbara Franke, Wilbert HM Peters, Johannes G van der Hoeven, Paul Smits, Peter Pickkers

**Affiliations:** 1 Department of Pharmacology-Toxicology, Radboud University Nijmegen Medical Center, Geert Grooteplein 10, 6500 HB, Nijmegen, The Netherlands; 2 Department of Intensive Care Medicine, Radboud University Nijmegen Medical Center, Geert Grooteplein 10, 6500 HB, Nijmegen, The Netherlands; 3 Department of Internal Medicine, Radboud University Nijmegen Medical Center, Geert Grooteplein 10, 6500 HB, Nijmegen, The Netherlands; 4 Department of Human Genetics, Radboud University Nijmegen Medical Center, Geert Grooteplein 10, 6500 HB, Nijmegen, The Netherlands; 5 Department of Gastroenterology, Radboud University Nijmegen Medical Center, Geert Grooteplein 10, 6500 HB, Nijmegen, The Netherlands

## Abstract

**Introduction:**

Preclinical studies have shown that the endogenous nucleoside adenosine prevents excessive tissue injury during systemic inflammation. We aimed to study whether endogenous adenosine also limits tissue injury in a human in vivo model of systemic inflammation. In addition, we studied whether subjects with the common 34C > T nonsense variant (rs17602729) of adenosine monophosphate deaminase (*AMPD1*), which predicts increased adenosine formation, have less inflammation-induced injury.

**Methods:**

In a randomized double-blinded design, healthy male volunteers received 2 ng/kg E. Coli LPS intravenously with (*n *= 10) or without (*n *= 10) pretreatment with the adenosine receptor antagonist caffeine (4 mg/kg body weight). In addition, lipopolysaccharide (LPS) was administered to 10 subjects heterozygous for the *AMPD1 *34C > T variant.

**Results:**

The increase in adenosine levels tended to be more pronounced in the subjects heterozygous for the *AMPD1 *34C > T variant (71 ± 22%, *P*=0.04), compared to placebo- (59 ± 29%, *P*=0.012) and caffeine-treated (53 ± 47%, *P*=0.29) subjects, but this difference between groups did not reach statistical significance. Also the LPS-induced increase in circulating cytokines was similar in the LPS-placebo, LPS-caffeine and LPS-AMPD1-groups. Endotoxemia resulted in an increase in circulating plasma markers of endothelial activation [intercellular adhesion molecule (ICAM) and vascular cell adhesion molecule (VCAM)], and in subclinical renal injury, measured by increased urinary excretion of tubular injury markers. The LPS-induced increase of these markers did not differ between the three groups.

**Conclusions:**

Human experimental endotoxemia induces an increase in circulating cytokine levels and subclinical endothelial and renal injury. Although the plasma adenosine concentration is elevated during systemic inflammation, co-administration of caffeine or the presence of the 34C > T variant of *AMPD1 *does not affect the observed subclinical organ damage, suggesting that adenosine does not affect the inflammatory response and subclinical endothelial and renal injury during human experimental endotoxemia.

**Trial Registration:**

ClinicalTrials (NCT): NCT00513110.

## Introduction

Sepsis, the systemic inflammatory response syndrome that occurs during infection, is associated with considerable morbidity and mortality in non-cardiac intensive care units [1]. During sepsis, the initial inflammatory response can be overwhelming, leading to significant collateral damage to normal tissues.

During systemic inflammation, the extracellular concentration of the endogenous nucleoside adenosine increases rapidly [2,3], with concentrations increasing up to 10-fold in septic shock patients [2]. Animal studies have shown that subsequent stimulation of adenosine receptors, mainly the adenosine A_2A _receptor, on various immune cells potently reduces the inflammatory response [4,5]. In humans, however, evidence that adenosine can limit the inflammatory response or prevent tissue injury is limited [6].

Interestingly, a genetic loss-of-function variant of the enzyme adenosine monophosphate deaminase (*AMPD1*) was recently shown to improve prognosis in patients with coronary artery disease [7], most likely because of augmented adenosine formation during ischemia in these patients [8]. It is unknown whether subjects with this polymorphism have an altered immune response or whether these individuals are protected from inflammation-induced organ injury.

**Figure 1:**
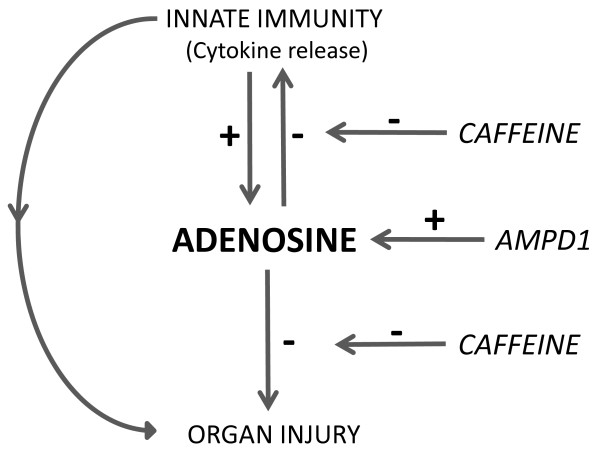
**Schematic view of the hypothesis**. During systemic inflammation, the circulating adenosine concentration increases rapidly, resulting in a negative feedback loop limiting (a) inflammation-induced cytokine release and (b) tissue injury. However, in the presence of caffeine, a non-selective adenosine receptor antagonist, this mechanism of protection is lost and inflammation-induced tissue damage will be aggravated. In the presence of the 34C > T variant of the *AMPD1 *gene, the inflammation-induced increase in adenosine concentration is augmented, and therefore the inflammatory response and organ injury are reduced. AMPD1, adenosine monophosphate deaminase.

In the present study, we addressed three major questions, illustrated in Figure 1. First, does systemic inflammation induced by experimental human endotoxemia increase the circulating adenosine concentration *in vivo*? Second, does this enhanced increase in circulating adenosine modulate the innate immune response? Third, does this increase reduce end-organ damage? We addressed these questions in healthy volunteers after systemic administration of lipopolysaccharide (LPS) with or without concomitant administration of the adenosine receptor antagonist caffeine. In addition, we separately studied healthy volunteers with the 34C > T variant of the *AMPD1 *gene to test the third hypothesis (that is, that the inflammation-induced increase in circulating adenosine is augmented and organ damage is attenuated in these subjects).

## Materials and methods

### Healthy volunteers

This study is registered at the ClinicalTrials.gov registry under the number NCT00513110. After the study was approved by the local ethics committee of the Radboud University Nijmegen Medical Centre, 43 healthy male volunteers provided written informed consent. Since the inflammatory response that occurs in this particular model is different in females [9], we included male subjects only. All volunteers had a normal physical examination, electrocardiography, and routine laboratory values before the start of the experiment. Since the prevalence of the *AMPD1 *SNP (single-nucleotide polymorphism) in Caucasian and African-American individuals is approximately 15% to 20%, we screened a total of 43 individuals. After genotyping of the *AMPD1 *rs17602729 variant (also known as 34C > T and Cys12Arg), we selected 10 subjects with the heterozygous (CT) genotype. Of the remaining 33 subjects, 20 subjects (at random) were asked by an independent research nurse to participate in the study and were randomly assigned to either the control or caffeine-treated group. Since the study was double-blind, the investigators who were involved in the conduct of the study were not aware of whether the patient belonged to the AMPD1 group or the caffeine or placebo group (both without the AMPD1 polymorphism).

Volunteers were asked not to take any prescription drugs, and they refrained from caffeine intake 48 hours prior to the LPS administration. The subjects were admitted to our clinical research unit on the day of the experiment and were kept under close observation for 10 hours.

### Experimental protocol

During the experiment, all volunteers were monitored for heart rate (electrocardiogram), blood pressure (intra-arterially), and body temperature (infrared tympanic thermometer; Sherwood Medical, 's-Hertogenbosch, The Netherlands) from 2 hours before the administration of LPS until the end of the experiment (8 hours after the LPS administration). A cannula was inserted in a deep forearm vein for prehydration (1.5 L of 2.5% glucose/0.45% saline solution in the hour before LPS administration) and LPS infusion. During the first 6 hours after the LPS administration, all subjects received 150 mL/hour and, after that period until the end of the experiment, 75 mL/hour of 2.5% glucose/0.45% saline solution to ensure an optimal hydration status [10].

An intra-arterial cannula was placed in the a. brachialis of the non-dominant arm, into which LPS was injected at t = 0 hours. The course of symptoms (headache, nausea, shivering, and muscle and back pain) was scored on a 6-point Likert scale (0 = no symptoms, 5 = very severe symptoms), resulting in a total score of 0 to 25. Blood was collected at various time points after LPS administration. Furthermore, during the first 10 minutes of every hour after LPS administration, forearm blood flow was determined in both forearms with venous occlusion plethysmography (Filtrass; DOMED Medizintechnik GmbH, Munich, Germany) as previously described [11,12].

The 20 subjects with the *AMPD1 *CC genotype (*n *= 20) received either caffeine (4 mg/kg body weight intravenously over 10 minutes [13]) or saline 10 minutes before LPS infusion. Caffeine, dosed at 4 mg/kg, has been shown to effectively antagonize the hemodynamic effects of adenosine, which are mediated by adenosine A_2A _receptor stimulation [14]. The 10 subjects heterozygous for the *AMPD1 *polymorphism (CT genotype) also received saline in a double-blinded fashion 10 minutes before LPS infusion.

### Endotoxin

US Reference *E. coli *endotoxin (*Escheria coli *O:113; Clinical Center Reference Endotoxin, National Institutes of Health, Bethesda, MD, USA) was used in this study. Ec-5 endotoxin, supplied as a lypophilized powder, was reconstituted in 5 mL of 0.9% saline for injection and vortex-mixed for at least 10 minutes after reconstitution. The endotoxin solution was administered as an intravenous bolus injection at a dose of 2 ng/kg of body weight.

### Blood collection for adenosine measurement

The circulating adenosine concentration was measured prior to and serially after the administration of LPS, as previously described [15]. With a special syringe system, the blood was immediately mixed with a 2.5-mL solution containing pharmacological blockers of adenosine formation, transport, and degradation immediately at the tip of the syringe. After blood was mixed with the 'blocker solution' and collected in the collection syringe with a total volume of 5 mL, the hematocrit value was determined in the mixture as a measure for dilution. Afterward, blood samples were centrifuged for 10 minutes at 1,000 *g *at 4°C and blood plasma was stored at -80°C until analyses.

The 'blocker solution' used to inhibit adenosine metabolism consisted of 40 μM dipyridamole (adenosine transport inhibitor), 10 μM erythro-9-(2-hydroxy-3-nonyl) adenine (EHNA) (adenosine deaminase inhibitor), 10 μM iodotubericidine (ITU) (adenosine kinase inhibitor), 13.2 mM Na_2_EDTA (disodium ethylenediamine tetraacetate) (inhibits release from platelets and acts as a 5ʹ-nucleotidase inhibitor), 118 mM NaCl, and 5 mM KCl.

### Genetic analysis

Blood was drawn in EDTA-containing vacutainers and stored at -80°C until DNA isolation. Genomic DNA isolation was performed with a standard desalting protocol [16]. Genotyping was performed by pyrosequencing according to the protocol of the manufacturer (Pyrosequencing AB, now part of Qiagen GmbH, Hilden, Germany) [17], as previously described [8].

### Determination of cytokines and adhesion molecules

Adhesion molecules ICAM (intercellular adhesion molecule) and VCAM (vascular cell adhesion molecule), indicators of shedding from the endothelium, were used as markers of endothelial dysfunction. To determine the concentration of the various cytokines and adhesion molecules, plasma was processed immediately by centrifugation at 2,000 *g *at 4°C for 15 minutes and stored at -80°C until analyses. Cytokine concentrations of tumor necrosis factor-alpha (TNF-α), interleukin (IL)-6, IL-1-receptor antagonist (IL1RA), and IL-10 were measured in samples taken at baseline and at 30, 60, 120, 240, and 480 minutes after LPS administration and subsequently analyzed batch-wise with a Luminex assay (Luminex Corporation, Austin, TX, USA) [18].

### Urine collection

Subjects collected urine in the 24 hours prior to the experiment. During the experiment, urine was collected 2 hours prior to LPS administration, the first 3 hours after LPS infusion, and between 3 and 8 hours after LPS infusion. During the sampling period, urine was kept on ice. Urine was processed, and GSTA1-1 (glutathione S-transferase alpha 1-1) and GSTP1-1 (glutathione S-transferase pi 1-1), as markers of proximal and distal tubular injury, respectively, were measured as previously described [19].

### Statistical analysis

Data with a Gaussian distribution were tested for significance by using repeated measures analysis of variance (ANOVA). Non-parametric data were analyzed with the Friedman test. The percentage increase in adenosine concentrations and increase in GSTA1-1 and GSTP1-1 were analyzed with the paired Student *t *test. Since most of the data had a non-Gaussian distribution, data are expressed as median (interquartile range [IQR]) unless specified otherwise. A *P *value of less than 0.05 was considered statistically significant.

## Results

### Baseline characteristics

Demographic characteristics did not significantly differ between the three groups of healthy volunteers (Table 1).

**Table 1:** Demographic characteristics

	Experimental endotoxemia		
	
Parameters	Placebo (*n *= 10)	AMPD1 (*n *= 10)	Caffeine (*n *= 10)
Age, years	23 (22-24)	23 (21-25)	22 (20-25)
Males/Females	10/0	10/0	10/0
Body mass index, kg/m^2^	21 (20-23)	23 (22-24)	22 (21-24)

Data for age and body mass index are presented as median (interquartile range). AMPD1, adenosine monophosphate deaminase.

### Changes in clinical, inflammatory, and hemodynamic parameters during human endotoxemia

**Figure 2:**
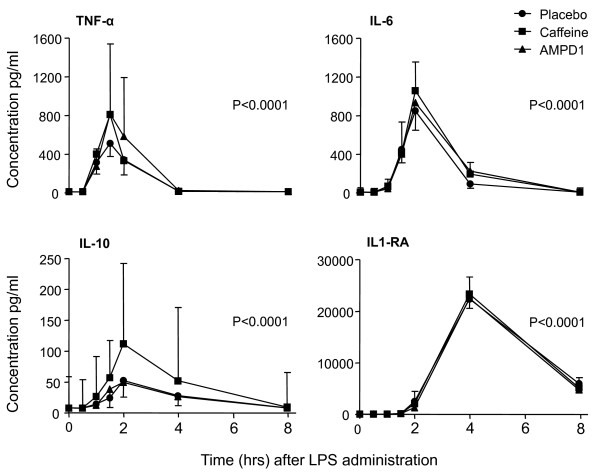
**Inflammatory parameters in the three groups (*n *= 10 per group)**. Administration of lipopolysaccharide (LPS) resulted in a marked increase in pro- and anti-inflammatory cytokines. Data are expressed as median [nterquartile range]) and were analyzed with one-way analysis of variance (ANOVA). The probability values refer to the significant increase in circulating cytokines for each group, as analyzed with repeated measures ANOVA. There was no significant difference between groups. AMPD1, adenosine monophosphate deaminase; IL, interleukin; IL1RA, interleukin-1-receptor antagonist; TNF-α, tumor necrosis factor-alpha.

In the 30 healthy volunteers, LPS administration induced the expected influenza-like symptoms, such as headache, nausea, and chills, starting after 60 to 120 minutes. The symptoms were mild, and all volunteers were symptom-free within 8 hours after LPS administration. Peak symptoms occurred approximately 90 minutes after LPS infusion. Body temperature was significantly elevated, with a peak temperature approximately 4 hours after LPS infusion (*P *< 0.0001, repeated measures ANOVA for each group), and white blood cell count decreased 1 hour after LPS administration, after which there was an increase with a peak 8 hours after LPS administration (*P *< 0.0001, repeated measures ANOVA for each group) (Table 2). Plasma concentrations of pro- and anti-inflammatory cytokines (TNF-α, IL-6, IL-10, and IL1RA) are shown in Figure 2. Thus, caffeine administration and the presence of the 34C > T variant of the *AMPD1 *gene did not change the inflammatory response to LPS.

**Table 2:** Clinical parameters and forearm blood flow response during human endotoxemia in the absence and presence of caffeine or the AMPD1 polymorphism

		T = 0	T = 1	T = 2	T = 4	T = 8
Δ Temperature, °C	Placebo	0.0 ± 0.0	0.3 ± 0.1	1.0 ± 0.1	1.3 ± 0.1	0.6 ± 0.1
	AMPD1	0.0 ± 0.0	0.3 ± 0.1	1.0 ± 0.2	1.6 ± 0.2	0.9 ± 0.1
	Caffeine	0.0 ± 0.0	0.3 ± 0.2	0.9 ± 0.2	1.6 ± 0.2	1.0 ± 0.2
Leukocytes, × 10^9^/L	Placebo	5.2 ± 0.8	3.0 ± 0.6	5.7 ± 0.6	8.9 ± 0.5	11.0 ± 0.5
	AMPD1	5.1 ± 0.4	2.3 ± 0.2	6.4 ± 0.9	9.6 ± 1.1	11.9 ± 1.1
	Caffeine	4.7 ± 0.3	2.4 ± 0.3	5.9 ± 0.7	10.6 ± 0.7	12.7 ± 0.7
FBF, mL/minute per dL forearm volume	Placebo	2.8 (2.6-5.6)	5.3 (3.2-6.9)	3.8 (2.5-4.7)	7.3 (6.2-8.6)	6.4 (4.3-7.6)
	AMPD1	3.1 (2.8-3.9)	3.1 (2.8-5.5)	3.0 (2.3-3.7)	6.2 (4.0-10.6)	5.8 (5.3-6.7)
	Caffeine	2.9 (2.1-3.5)	3.9 (3.1-4.7)	2.6 (2.2-3.0)	7.9 (5.3-10.7)	6.7 (5.7-7.4)

Lipopolysaccharide-induced changes were significant (*P *< 0.001, repeated measures analysis of variance) for each group but not significantly different between groups. Data are presented as mean ± standard error of the mean. Forearm blood flow (FBF) data are presented as median (interquartile range) since FBF data had a non-Gaussian distribution. AMPD1, adenosine monophosphate deaminase.

**Figure 3:**
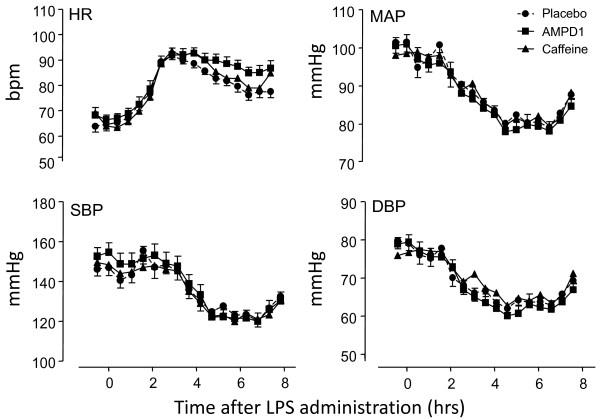
**Hemodynamic profile in response to endotoxemia (mean ± standard error of the mean, *n *= 10 subjects per group)**. Lipopolysaccharide (LPS) administration resulted in an increase in heart rate (HR) and decreases in mean arterial pressure (MAP), systolic blood pressure (SBP), and diastolic blood pressure (DBP) for each group (*P *< 0.01 repeated measures analysis of variance). There was no significant difference between groups. AMPD1, adenosine monophosphate deaminase; bpm, beats per minute.

LPS administration induced a decrease in blood pressure and an increase in heart rate (Figure 3). There were no significant differences in hemodynamic parameters and plasma cytokine levels between the three experimental groups. Forearm blood flow increased during experimental human endotoxemia, with a maximal response 4 hours after LPS administration (Table 2).

### The effect of lipopolysaccharide infusion on the endogenous adenosine concentration

**Figure 4:**
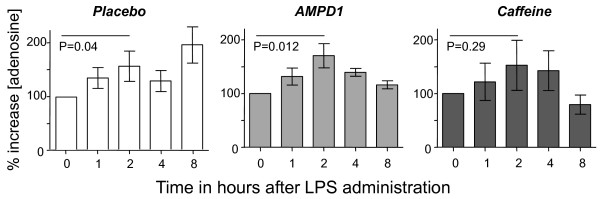
**Percentage increase in plasma adenosine concentration after lipopolysaccharide (LPS) administration for each group**. Data are expressed as mean ± standard error of the mean. Data were analyzed with the paired Student *t *test. There were no significant differences between groups. AMPD1, adenosine monophosphate deaminase.

The increase in adenosine levels tended to be more pronounced in the subjects heterozygous for the *AMPD1 *34C > T variant (from 9.0 [IQR 8.5 to 11.5] at baseline to 16.5 [11.8 to 21.5] ng/mL 2 hours after LPS infusion, an increase of 71% ± 22%; *P *= 0.04) compared with the placebo group (from 10.0 [IQR 8.8 to 13.0] at baseline to 14.0 [12.3 to 19.0] ng/mL, an increase of 59% ± 29%; *P *= 0.012), but this difference between groups did not reach statistical significance. In the caffeine-treated subjects, the adenosine concentration increased from 12.0 [IQR10.0 to 18.0] at baseline to 18.0 [12.5 to 32.5] ng/mL, an increase of 53% ± 47% (*P *= 0.29). Figure 4 illustrates the LPS-induced changes in circulating adenosine.

Caffeine levels in the placebo and *AMPD1 *34C > T groups did not exceed 0.08 mg/mL either before or after LPS infusion. In the caffeine group, caffeine levels were 0.04 [0.02 to 0.06] at baseline and 6.0 [5.6 to 6.4] mg/mL 1 hour after caffeine infusion (*n *= 10).

### The effect of lipopolysaccharide infusion on end-organ injury

#### Vascular dysfunction

**Figure 5:**
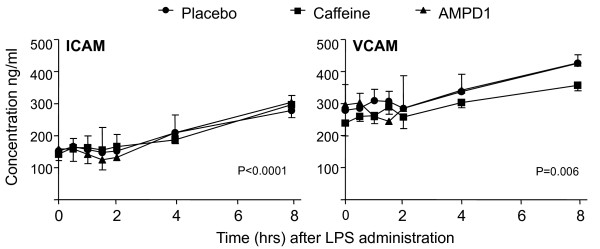
**Administration of lipopolysaccharide (LPS) resulted in a marked increase of intercellular adhesion molecule (ICAM) and vascular cell adhesion molecule (VCAM), markers of endothelial activation**. Data are expressed as median [interquartile range]. The probability values refer to the significant increase in circulating adhesion molecules for each group, as analyzed with repeated measures analysis of variance. No significant difference between groups was found. AMPD1, adenosine monophosphate deaminase.

Plasma levels of ICAM and VCAM, markers of endothelial function, increased following LPS administration (Figure 5) (*P *< 0.0001 for ICAM and *P *= 0.006 for VCAM, ANOVA repeated measures). There was no significant difference in the LPS-induced increase in plasma ICAM and VCAM concentrations between the three groups (*P *> 0.1).

#### Renal injury

**Figure 6:**
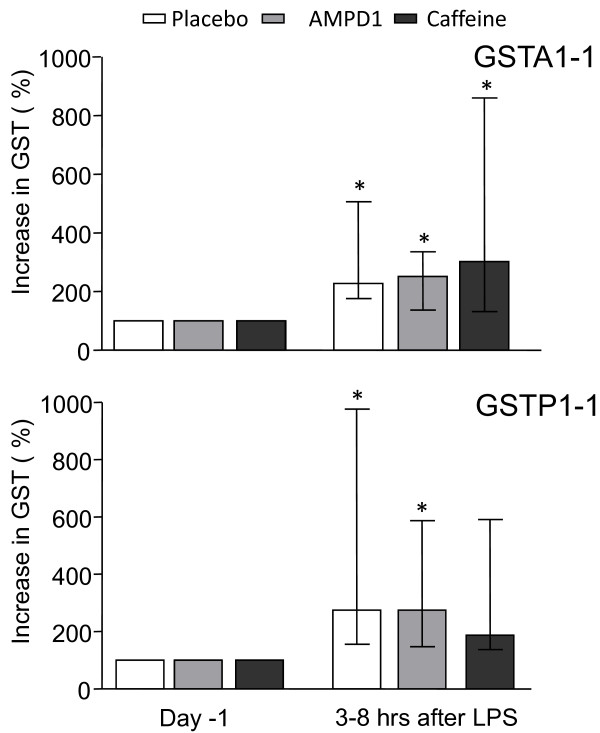
**Excretion of glutathione-S-transferases (GSTs) in urine**. Administration of lipopolysaccharide (LPS) resulted in a marked increase in the urinary excretion of markers of proximal and distal tubular damage. Data are expressed as percentage increase in time after LPS infusion (median [interquartile range]). Data were tested with a paired Student *t *test. *P < 0.05. No significant difference between groups was found. AMPD1, adenosine monophosphate deaminase; GSTA1-1, glutathione S-transferase alpha 1-1; GSTP1-1, glutathione S-transferase pi 1-1.

Glutathione-S-transferases (GSTs) are *cytosolic *enzymes that are present in the cells of the proximal tubule (GSTA1-1) and distal tubule (GSTP1-1). A very low urinary excretion rate is present during physiological circumstances. Both GSTA1-1 and GSTP1-1 levels, respectively, increased during experimental endotoxemia (Figure 6) (*n *= 30, *P *< 0.0001). There were no differences between the LPS-induced increase in the three experimental groups (*P *> 0.2).

## Discussion

In the present study, we show for the first time that acute systemic inflammation induced by human experimental endotoxemia results in an increase in circulating endogenous adenosine in humans *in vivo*. Apparently, the systemic inflammatory response during experimental endotoxemia is sufficient to stress the body to a level that induces adenosine release. These results are in accordance with those of previous findings demonstrating increased plasma adenosine concentrations in humans with septic shock [2,3,20]. We found no evidence that circulating adenosine exerted immune modulatory effects or tissue-protective effects during inflammation. Pretreatment with the adenosine receptor antagonist caffeine did not potentiate the inflammatory response or the inflammation-induced subclinical organ damage, suggesting that this increased adenosine concentration does not act as a negative feedback signal to temper inflammation and organ damage in this model.

Previous *in vitro *and animal studies have provided robust evidence that endogenous adenosine plays a pivotal role in the limitation of excessive tissue injury in situations of inflammation, mainly by activation of adenosine A_2A _receptor [5]. In humans *in vivo*, however, data on the effect of inflammation on the endogenous adenosine concentration are limited to only one small study in which the plasma adenosine concentration was significantly higher in patients with septic shock compared with control patients [2].

In this study, we studied the effect of inflammation on circulating adenosine in a well-validated model of systemic inflammation [21] and used a previously described method to measure the plasma adenosine concentration [15]. Our results show that, during endotoxemia, the endogenous adenosine concentration increases in time, with a maximum concentration reached 2 hours after LPS administration. Recently, measuring circulating adenosine in 10 septic shock patients who were admitted to the intensive care unit, we found a median (IQR) adenosine concentration of 30.9 [24.1 to 39.8] ng/mL (BPR, NPR, PvdB, JGvdH, PS, and PP, unpublished observations). The adenosine concentration was lower in the LPS-treated volunteers, probably indicating that the less severe and shorter duration of the inflammatory response during experimental endotoxemia induces a smaller insult compared with septic shock. In addition, in septic shock patients, not only the inflammatory response but also tissue hypoperfusion may play a role in the formation of adenosine. We subsequently aimed to demonstrate that this increased circulating adenosine could act as a negative feedback molecule, which attenuates the inflammatory response and ameliorates end-organ dysfunction. To this end, subjects were pretreated with the nonselective adenosine receptor antagonist caffeine [22] in a dose previously shown to completely block the cardiovascular effects of adenosine [13]. Subjects were asked to refrain from caffeine ingestion for the 48-hour period prior to the experiment in order to reveal any effects of adenosine receptor stimulation [23]. At the moment of LPS administration, the plasma caffeine concentration averaged 6.0 mg/L, which is a concentration previously shown to effectively antagonize adenosine receptor stimulation [14,24]. In more detail, we recently showed that an intravenous dose of caffeine of 4 mg/kg, similar to the dose of the present study, completely blunted ischemic preconditioning, which is mediated by adenosine receptor stimulation [13]. In addition, our group has demonstrated, in the past, that caffeine in a plasma concentration of 5 mg/L significantly antagonizes the hemodynamic effects of adenosine administration [14].

Previous studies in animal models have shown that caffeine is able to potentiate the production of pro-inflammatory cytokines both *in vitro *[25,26] and *in vivo *[27] and that caffeine exacerbates tissue injury during inflammation [5,24]. In contrast to these results, in our human endotoxemia model, caffeine did not augment the immune response nor did it increase (subclinical) organ damage. There are several potential explanations for this finding. First, endogenous adenosine may not have an important anti-inflammatory potential in humans *in vivo*. However, this is not likely, given the consistent findings in animal studies and isolated cell studies and given the observation that administration of exogenous adenosine can limit the IL-6 response during human experimental endotoxemia [6]. Second, the limited increase in adenosine in our model might not be sufficient to induce significant anti-inflammatory effects. Recently, Soop and colleagues [28] demonstrated that the administration of 40 μg/kg per minute adenosine attenuated the release of the soluble RAGE (receptor for advanced glycation end products) but was unable to decrease the pro-inflammatory response. Unfortunately, no endogenous adenosine concentrations were measured in that study, although it was speculated that blood adenosine levels were at the submicromolar range. Finally, it needs to be realized that caffeine only blocks the adenosine A_1_, A_2A_, and A_2B _receptors in the dose we used. Therefore, stimulation of the adenosine A_3 _receptor, which also exerts anti-inflammatory potential, may have counteracted the pro-inflammatory effects of caffeine [29-31]. Specific adenosine subtype receptor antagonists are being developed but are not currently available for human use.

We studied the effect, in a separate group of healthy volunteers, of the common 34C > T variant of the *AMPD1 *gene on the adenosine concentration and subclinical end-organ damage during endotoxemia. In Caucasians, approximately 20% of subjects are heterozygous for this variant allele, encoding a premature stopcodon, which results in a dysfunctional enzyme [32]. AMPD catalyzes the intracellular conversion of AMP into IMP (inosine monophosphate). Subjects heterozygous for this variant allele appear to have a 50% reduction in enzyme activity [33]. Interestingly, heterozygosity was recently associated with an improved cardiovascular prognosis in patients with coronary artery disease, probably because of an increased conversion of AMP into adenosine with subsequent increased adenosine concentrations and subsequent organ protection during ischemia [8]. Considering the beneficial cardiovascular effects of adenosine receptor stimulation in subjects with AMPD deficiency [7,34], we hypothesized that endotoxemia-induced adenosine formation and subsequent adenosine receptor stimulation would also be potentiated. Although the LPS-induced increase in adenosine concentrations tended to be most strongly potentiated in the *AMPD1 *heterozygous group (with a mean increase of 71% versus 59% and 53% in the placebo and caffeine groups, respectively), this difference between groups did not reach statistical significance. Moreover, we did not observe an attenuation of organ damage in subjects heterozygous for the *AMPD1 *variation. A different route of adenosine formation during inflammation as compared with situations of ischemia could be an explanation. During ischemia/hypoxia, an increased intracellular degradation of ATP significantly contributes to the increase in extracellular adenosine. In this situation of increased intracellular AMP availability, a reduction of AMPD activity could have an important effect on adenosine formation. In contrast, during inflammation, the main source of adenosine formation following endotoxemia is the extracellular hydrolysis of ATP instead of an intracellular increase in AMP. Previous studies have suggested that inflammation directly leads to active release of adenine nucleosides, such as ATP, as well as passive release due to endothelial cell damage [35]. ATP is then quickly converted into adenosine. During sepsis, tissue hypoxia will most likely also play an important role in the accumulation of adenosine [36,37]; however, this is unlikely during the relatively mild model of experimental endotoxemia. This could explain why the *AMPD1 *polymorphism did not influence the inflammation-induced increase in extracellular adenosine concentration. The lack of a significantly more pronounced increase in circulating adenosine in *AMPD1 *subjects may also be explained by the fact that adenosine is produced locally in the tissue and the endothelium acts as an active metabolic barrier for adenosine. Thus, circulating adenosine concentrations may not correctly reflect the inflammation-induced adenosine increase in the interstitial compartment. Pharmacological interventions, such as dipyridamole, an adenosine re-uptake inhibitor that increases the local adenosine concentration [38], or pentoxifylline, of which the immunomodulatory effects depend on sufficient levels of adenosine [20], may represent new therapeutic interventions to modulate the immune response.

## Conclusions

Human experimental endotoxemia results in systemic inflammation and increases the circulating endogenous adenosine concentration. Pharmacological blockade of the adenosine receptors, however, does not augment the innate immune response or its resultant (subclinical) organ injury. In addition, organ damage is not reduced in subjects with the AMPD1 polymorphism, despite the tendency to a more pronounced LPS-induced increase in endogenous adenosine in these subjects. Given these observations, we conclude that, during human endotoxemia, endogenous adenosine does not act as a negative feedback molecule to limit the inflammatory response and subsequent tissue injury.

## Key messages

• During human experimental endotoxemia (as a model of systemic inflammation), the circulating adenosine concentration increases.

• Blockade of the adenosine receptor with caffeine does not augment the inflammatory response or subsequent organ damage.

• The presence of the *AMPD1 *polymorphism is associated with increased levels of adenosine but does not affect the inflammatory response during human experimental endotoxemia.

• We conclude that the slight increase in endogenous adenosine that occurs during human endotoxemia is not sufficient to act as a negative feedback mechanism to control the inflammatory response.

## Abbreviations

AMPD1: adenosine monophosphate deaminase; ANOVA: analysis of variance; GSTA1-1: glutathione S-transferase alpha 1-1; GSTP1-1: glutathione S-transferase pi 1-1; ICAM: intercellular adhesion molecule; IL: interleukin; IL1RA: interleukin-1-receptor antagonist; IQR: interquartile range; LPS: lipopolysaccharide; TNF-α: tumor necrosis factor-alpha; VCAM: vascular cell adhesion molecule.

## Competing interests

The authors declare that they have no competing interests.

## Authors' contributions

BPR carried out the study, gathered all data, performed the statistical analysis and wrote the manuscript. PvdB performed the adenosine and caffeine measurements. BF supervised the genetic analyses and the writing of the manuscript. WHMP performed the GSTA1-1 and GSTP1-1 analyses. PP, NPR, and PS supervised the conduct of the study and the writing of the paper. JGvdH corrected the manuscript. All authors read and approved the final manuscript.
